# Problems in the Estimation of the Key Parameters using MLE in Lung Cancer Screening

**Published:** 2020-08-21

**Authors:** Dongfeng Wu, Seongho Kim

**Affiliations:** 1 Department of Bioinformatics and Biostatistics, University of Louisville, Louisville, KY 40202; 2 Biostatistics Core, Karmanos Cancer Institute, Department of Oncology, School of Medicine Wayne State University Detroit, Michigan 48201

This is a short review article on the problems that we have encountered in the parameter estimation using likelihood functions and MLE in lung cancer screening. We also provide some ideas on how to fix these problems in practice.

We have been working in the area of cancer screening modeling for many years. A well-known and frequently used model in cancer screening is the progressive three-state model [1], where all cancer patients are assumed to go through three states: the disease-free state when one is cancer-free or the cancer is in an early stage that no technology can find; the preclinical state when one without symptom but cancer could be detected by screening, and the clinical state when cancer related symptoms show up. There are three key parameters in the model: a) the screening sensitivity, the probability of a positive screening result given that one is in the preclinical state; b) the distribution of sojourn time, which measures the time duration in the preclinical state; and c) the transition density, which measures the time duration in the disease-free state, or the onset age of the preclinical state. These three parameters are called the key parameters since they determines the screening processes and all other terms, for example, lead time (diagnosis time advanced by screening), probability of over-diagnosis, etc., are functions of these three. Therefore accurate estimation of these three key parameters is critical and lays a foundation for all other estimations.

A commonly used method to estimate the three key parameters is to use a likelihood function and the maximum likelihood estimate (MLE) [2-7]. However, when the number of the screening is less than four, it is very hard for the MLE to be close to the true value of the input parameters in any single run based on our simulation study. What we did was: step 1, generate pseudo screening data using some fixed input parameters; step 2, using the generated pseudo data and the likelihood to calculate the MLE; step 3, repeat the first two steps N (≥ 200) times, and compare the average of the MLE with the input parameters, and measure the errors in terms of the mean and the standard deviation. It turns out that unless a large sample size (such as 10^5^) for each age group is used and unless the number of screening is at least 4, the average of the estimate MLE won’t be very close to the true input value; In the case of the large sample size, even though the average of MLE is close to the true input values, it still has a large standard error. Hence, for a single collected screening dataset, it is very hard to say whether the MLE is close to the true parameters.

The major problems for using the likelihood function and its MLE is: it is often a plateau-shaped function of the parameters with many local maxima (or minima), especially for sensitivity. In another word, a deterministic method to find the maximum (or minimum if you use the negative of the likelihood) is not sensitive enough. It depends on the initial values and very likely to find a local maximum (or minimum) after a finite iterations. To correct this, we have tried different initial values, developed different kinds of likelihoods, using conditional probabilities, hoping to improve it. However, the improvement is negligible.

This is what we have found: 1. Smaller sample size (population in each age group should be at least 10,000 in the simulation) cannot achieve much accuracy. In fact, standard deviation is much larger than the mean difference, showing that the variation is too large. In reality, sample size in each age group for existing screening program is much smaller. For example, in the recently finished National Lung Screening Trial (NLST) low dose CT arm, the largest two age groups has about 2600 participants each at initial screening, and it is usually less than 2000 in other age groups, with some of the age group has less than 500 participants. 2. Using conditional likelihood won’t improve the accuracy. Since there are many people dropped out of the screening program in the middle of the process, we thought maybe using conditional probability at each screening could handle the dropout problems better; the result is not significant, especially when the screening program only has 3 or fewer exams, such as in the NLST study. The reason may be due to the fact that since the screening number is fewer, and the screening interval is one year apart, the conditional probability of be asymptomatic before each screening is close to 1, making it the same as using the unconditional probability.

The most annoying problem is that the MLE estimate of screening sensitivity is over-estimated. This is especially obvious in the chest X-ray screening data in the PLCO [3] and the NLST study [4]. In the NLST study, the screening program composed of three annual exams, and there are two randomly assigned arms, chest X-ray and low-dose CT. Just looking at the screen-detected number and the interval-incident number, we can roughly estimate the sensitivity by dividing the screen- detected number with the total diseased cases (screen-detected cases plus interval cases), and know that low-dose CT has a much high sensitivity which is above 90%, while chest X-ray has a lower sensitivity between 60-70%. And due to the random assignment of participants, it is reasonable to assume that the two groups of participants share some common characteristics, such as both are heavy smokers, hence the estimated parameters regarding the sojourn time and the transition density should be close to each other. And the only difference should be in the screening sensitivity. However, it turns out, the estimated MLE of sensitivity from both groups are both close to 1, which is definitely untrue for the X-ray group. And we have to put some upper limit to get a reasonable estimate for the sensitivity for the X-ray group.

So how we are going to deal with these problems and get a reasonably accurate estimate of the key parameters? The best way is to use Bayesian posterior samples, and also put some reasonable limit or boundary on the sensitivity. A great advantage of the Bayesian posterior samples is that it is not focus on one specific point as an estimate, but reflects the posterior distribution of the sensitivity and its variation. It is also important to put a reasonable boundary for sensitivity based on the epidemiology result, so we can get the posterior samples of sensitivity in a reasonable range and reflects the true and not inflated distribution. Finally, a suggestion to future mass screening program investigators/designer or policy makers are: to apply at least 4 screenings, not three or less; otherwise, it is difficult to use the screening data and obtain meaningful information from it.
